# “Outcome measures in Iranian studies of carpal tunnel syndrome surgery: a systematic review”

**DOI:** 10.1186/s12893-026-03712-7

**Published:** 2026-04-27

**Authors:** Hesam Alitaleshi, Kamran Eftekhari, Leila Zanjani, Mohammad Reza Abbaszadeh, Mohammad Reza Dolikhani, Ghazaleh Shakibamaram

**Affiliations:** 1 Center for Orthopedic Trans-Disciplinary Applied Research, Tehran University of Medical Sciences, Tehran, Iran; 2 Department of Orthopedics, School of Medicine, Kermanshah University of Medical Sciences, Kermanshah, Iran; 3 Department of Orthopedic and Trauma Surgery, Shariati Hospital, Tehran University of Medical Sciences, Tehran, Iran; 4 Department of Orthopedics, Imam Hossein Medical Center, Shahid Beheshti University of Medical Sciences, Tehran, Iran; 5 Department of Orthopedics and Trauma Surgery, Shariati Hospital, North Kargar Ave, Jalal-e-Al-e-Ahmad Hw, Tehran, 14117 13135 Iran

**Keywords:** Carpal tunnel syndrome, Carpal tunnel release, Outcome measures, Patient-reported outcome measures, Boston Carpal Tunnel Questionnaire (BCTQ), Iran, Systematic review

## Abstract

**Background:**

Carpal tunnel syndrome (CTS) is the most prevalent peripheral nerve compression neuropathy. Its annual global incidence is approximately 2.3%, while prevalence estimates in Iran are around 17%. Surgery is typically recommended when conservative treatment fails or motor weakness is present. Despite numerous Iranian studies on CTS surgery, outcome assessment methods remain inconsistent. This systematic review aimed to evaluate the tools and methodologies used in Iranian research to report postoperative outcomes.

**Methods:**

Major databases were searched (1996–2025) for Iranian CTS surgery studies (≥ 3-week follow-up). We extracted study design, sample size, follow-up duration, surgical technique, and outcome measures. Outcome reporting was summarized using descriptive statistics (frequencies and percentages) and grouped into Patient-Reported Outcome Measures (PROMs), physical examination findings (e.g., grip strength), and electrodiagnostic parameters for structured comparison.

**Results:**

Twenty-two studies met the inclusion criteria. The Boston Carpal Tunnel Questionnaire was used in 45.5% of studies, pain was assessed with the Visual Analog Scale in 59.1%, and grip and pinch strength were evaluated in 40.9% and 13.6%, respectively. Electrodiagnostic testing was performed preoperatively in 81.8% of studies, but only 18% assessed postoperative changes. Reporting of return to work, scar length, and surgical technique was inconsistent.

**Conclusions:**

Iranian CTS research shows substantial variability in study design and outcome assessment. Although the volume of research is increasing, the methodological quality remains suboptimal, with a high prevalence of risk of bias in RCTs and poor quality in observational studies. The limited use of standardized instruments and inconsistent reporting may reduce comparability and clinical relevance.

**Supplementary Information:**

The online version contains supplementary material available at 10.1186/s12893-026-03712-7.

## Background

Carpal tunnel syndrome (CTS) is one of the most common conditions encountered in upper-extremity practice [1]. CTS is the most prevalent peripheral nerve entrapment neuropathy, and a recent meta-analysis estimated a global prevalence of approximately 14.4% [2].

In Iran, a systematic review and meta-analysis reported a pooled prevalence of approximately 17%; however, this estimate is derived mainly from cross-sectional data in heterogeneous populations [3]. It is estimated that over 500,000 carpal tunnel release surgeries are performed each year in the U.S., generating an annual healthcare cost of nearly two billion dollars [4].

Treatment of CTS includes both non-surgical and surgical approaches. Non-surgical options such as wrist splinting and corticosteroid injections are typically recommended in early stages [5]. However, when conservative treatments fail or an objective motor deficit is present, surgical intervention is generally advised.

The outcomes of carpal tunnel surgery are evaluated through various parameters, including electrophysiological assessments, measurements of pinch and grip strength, sensory and motor symptom evaluation, and patient-reported outcomes using standardized questionnaires [6].

Several studies have been conducted in Iran regarding carpal tunnel surgery. The aim of this study is to examine the evaluation criteria used in assessing surgical outcomes of CTS.

Standardized outcome reporting is fundamental for validating clinical results and enabling reliable comparisons between studies. The current lack of a unified assessment framework in Iranian orthopedic research limits the synthesis of regional data and its integration into global evidence. Therefore, this systematic review aimed to evaluate the outcome measures and methodologies utilized in Iranian research to provide a framework for improving the quality and comparability of future studies.

## Methods

### Protocol and registration

This systematic review was conducted in accordance with the Preferred Reporting Items for Systematic Reviews and Meta-Analyses (PRISMA) guidelines. The completed PRISMA 2020 checklist is provided as Supplementary Table S1. Although the protocol was not prospectively registered in PROSPERO (therefore, no amendments were made), the review process strictly adhered to pre-defined eligibility criteria to ensure methodological rigor and minimize selection bias.

### Search strategy

To evaluate the outcome measures utilized in assessing surgical outcomes of carpal tunnel syndrome (CTS) in Iran, a systematic literature search was conducted on June 17, 2025, across four major databases: PubMed, Embase, Scopus, and the Cochrane Library. Additionally, the first 10 pages (approximately 100 results) of Google Scholar were manually screened to identify potentially relevant studies not indexed in the core databases. To ensure the inclusion of high-quality data and facilitate international comparability, the search was restricted to English-language publications indexed in these global databases.

The search covered studies published between 1996 and 2025. Boolean search strategies were applied using combinations of relevant keywords. A sample search string used in PubMed was: (“carpal tunnel syndrome“[MeSH] OR “carpal tunnel“[tiab] OR “carpal tunnel syndrome“[tiab] OR “carpal tunnel release“[tiab] OR “carpal tunnel surgery“[tiab]) AND (“Iran“[tiab] OR “iran“[affiliation])

### Eligibility criteria

Studies were selected based on the following inclusion and exclusion criteria:

#### Inclusion criteria

1. Original research articles (randomized controlled trials (RCTs), cohort, cross-sectional, and case-series) conducted in Iran.
2. Studies focusing on surgical treatment of CTS (open, endoscopic, or other techniques).
3. Studies reporting postoperative clinical or functional outcomes.
4. A minimum follow-up duration of three weeks.
5. Full-text availability in English.

#### Exclusion criteria

1. Reviews, letters to the editor, and conference abstracts.
2. Case reports or studies with very small sample sizes (*n* < 10) lacking statistical power.
3. Studies focusing exclusively on non-surgical interventions (e.g., splinting, corticosteroid injection only).
4. Studies lacking clearly defined outcome assessment methodologies.

### Study selection

Two reviewers (H.A. and K.E.) screened the titles and abstracts of retrieved records to identify potentially relevant studies. Full-text articles of the selected records were subsequently retrieved and assessed against the eligibility criteria. Any discrepancies regarding study inclusion were resolved through discussion and consensus by consultation with a third reviewer (L.Z.).

### Data extraction

Two reviewers (H.A. and K.E.) independently extracted data. Discrepancies were resolved by a third reviewer (L.Z.) A structured data extraction form was developed to systematically collect the following variables from each eligible study. When study data were missing, incomplete, or unclear, we attempted to contact the corresponding authors by email to obtain clarification or additional information. If no response was received, the data were recorded as missing. The extracted data included:

#### Study characteristics

First author, publication year, study design, sample size, and follow-up duration.

#### Surgical details

Type of intervention (e.g., open vs. endoscopic), surgical technique, and anesthesia method.

#### Outcome measures

Usage of Patient-Reported Outcome Measures (PROMs) such as the Boston Carpal Tunnel Questionnaire (BCTQ) or DASH; physical examination findings (grip and pinch strength, sensory testing); pain assessment (Visual Analog Scale - VAS); and electrodiagnostic parameters.

#### Additional variables

Reporting of return-to-work time, scar length, and complications.

### Quality assessment

The methodological quality of the included studies was independently assessed by two reviewers (G.S. and M.D.). For RCTs, the Cochrane Risk of Bias tool (RoB 2) was employed to evaluate domains such as randomization, deviation from intended interventions, and missing outcome data. For non-randomized(Cohort and Case-Control) studies, the Newcastle-Ottawa Scale (NOS) was used. JBI Critical Appraisal Checklist was also used for Case series and Cross-Sectional studies. For any disagreements in quality scoring were resolved by consultation with a third reviewer (L.Z.).

### Data synthesis

Descriptive statistics (frequency and percentage) were used to summarize the utilization of different outcome measures. The extracted outcomes were synthesized and categorized into PROMs, physical examination findings, and electrodiagnostic parameters to facilitate structured comparison. Additionally, a temporal trend analysis was performed to evaluate changes in outcome reporting standards and the adoption of validated questionnaires over time. Effect measures were not calculated because no meta-analysis was performed, and outcomes were summarized descriptively as frequencies and percentages. No data conversions or imputations were performed. Certainty of evidence (e.g., GRADE) was not assessed because no quantitative synthesis was performed, and the review aimed to descriptively map outcome measures.

### Use of AI-assisted tool

During the preparation of this manuscript, the authors used ChatGPT (OpenAI; GPT-5 Thinking) and Grammarly (Grammarly, Inc.) on January 25, 2026, exclusively to improve grammar, spelling, punctuation, phrasing, and overall clarity of expression. All AI-assisted outputs were critically reviewed, verified, and edited by the authors, who assume full responsibility for the final content of the manuscript.

## Results

### Study selection

**Fig. 1:**
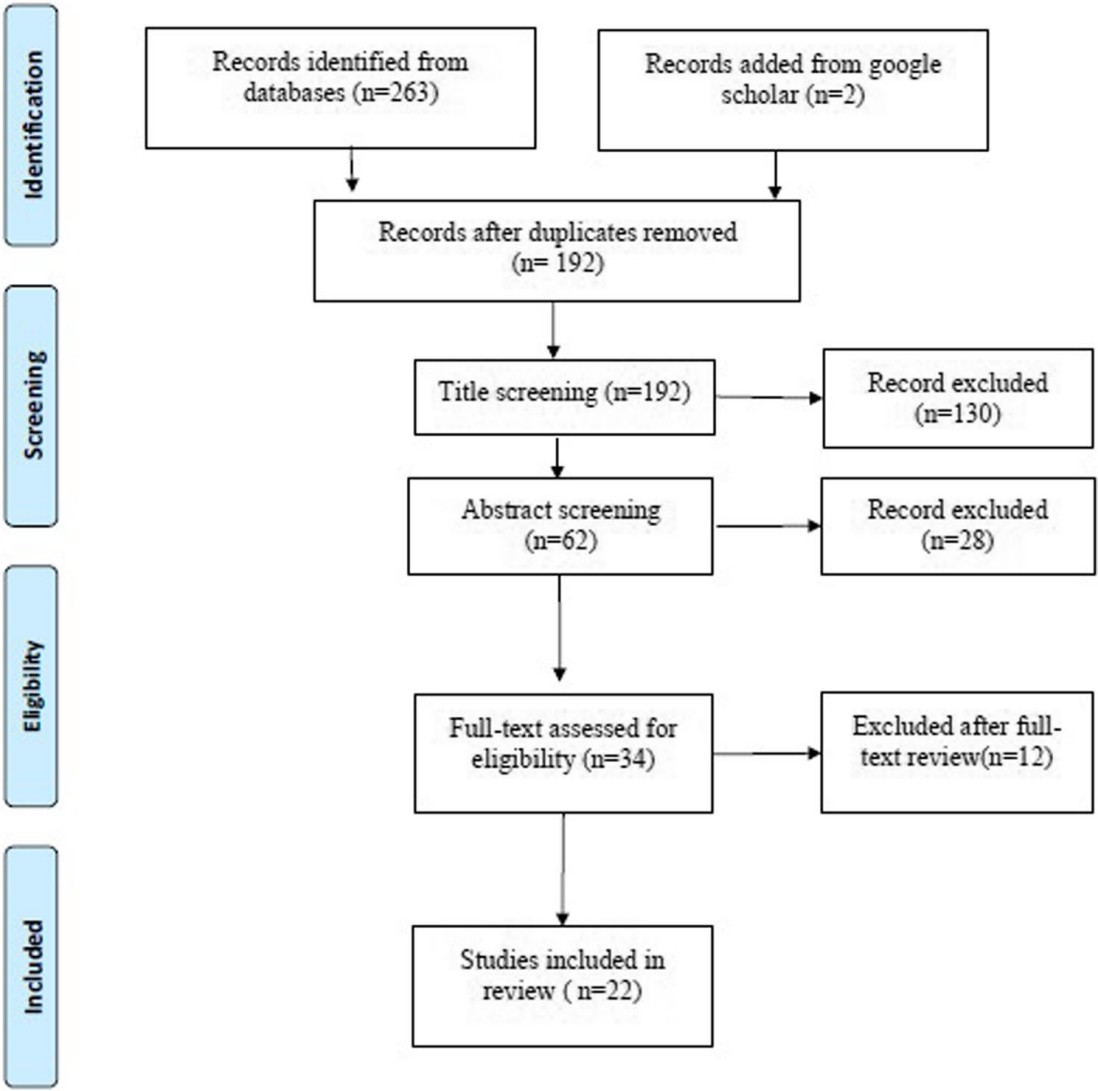
PRISMA flow diagram for screening prosses

A total of 263 records were initially identified through database searching, with an additional 2 records retrieved via manual screening. After removing 73 duplicates and screening titles and abstracts, 34 full-text articles were assessed for eligibility. During screening, 112 studies focusing on non-surgical treatments, 6 case reports, and 1 study lacking full-text access were excluded. Of these, 12 studies were excluded due to non-surgical interventions, insufficient outcome reporting, or lack of extractable data. Ultimately, 22 studies met the inclusion criteria and were included in the final analysis [7–28]. The complete study selection process is illustrated in Fig. 1 (PRISMA Flow Diagram)*.*

### Study characteristics

Among the included studies, 9 (40.9%) were RCTs, 8 were cohort studies, and the remainder comprised case-series, case-control, or cross-sectional designs. The mean follow-up duration was 5.27 months (SD = 3.76), ranging from 3 weeks to 18 months. The average sample size was 89 patients (range: 17–230) (Table 1)*.*

**Table 1:** Characteristics of included studies

Year	Author	Study type	Sample size (*N*)	Follow-up	Outcome Measures
2012	Aslani	RCT	105	4 months	Grip strength
2012	Tahririan	Case Series	17	6 months	NCS/EDX (DML/DSL/SCV)
2013	Ebrahimzadeh	Cohort	70	6 months	WHOQOL-BREEF; MHQ
2013	Heidarian	RCT	59	3 weeks	Pain (VAS)
2014	Nazerani	Case Series	176	12 months	Pain (VAS); Grip strength; Hand Questionnaire
2016	Sajjadi Saravi	RCT	45	3 months	Pain (VAS); Grip strength
2016	Roshanzamir	Case Control	47	3 weeks	BCTQ
2018	Abedi	Cohort	100	6 months	VAS, BCTQ
2019	Mottaghi	RCT	43	2 months	BCTQ
2019	Mardanpour	Case Series	188	18 months	Pain (VAS)
2020	Afshar	Cohort	70	6 months	BCTQ
2020	Alimohammadi	Cohort	152	6 months	BCTQ; Grip strength
2020	Khoshnevis	Cohort	75	5 months	Pain (VAS); Grip strength
2021	Razavipour	Cohort	60	6 months	BCTQ; QuickDASH; Pain (VAS); Pinch strength; Grip strength
2022	Daliri	Cross-sectional	26	3 months	BCTQ; Pain (VAS)
2022	Hajibarati	Cohort	46	6 weeks	Pain (VAS); Grip strength
2022	Khodadadi	Cohort	218	6 months	BCTQ; Grip strength
2022	Noorizadeh	RCT	105	6 months	BCTQ; Pain (VAS)
2023	Pahlevan Sabagh	RCT	60	6 months	Cosmetic outcomes (Scar width, height, pigmentation)
2024	Akhoondinasab	RCT	20	3 months	Pain (VAS); Modified Mayo Wrist Score
2024	Razavipour	RCT	230	6 months	BCTQ; Pain (VAS); Pinch strength; Grip strength
2024	Saeed-Banadaky	RCT	46	3 months	Pain (VAS); Pinch strength

“Not reported” refers to the absence of the specific outcome measures targeted in this review (BCTQ, VAS, Pinch, Grip) in the study

*Abbreviations*: *RCT* Randomized Controlled Trial, *NCS* Nerve Conduction Study, *EDX* Electrodiagnostic, *DML* Distal Motor Latency, *DSL* Distal Sensory Latency, *SCV* Sensory Conduction Velocity, *Quasi-Exp* Quasi-Experimental, *BCTQ* Boston Carpal Tunnel Questionnaire, *VAS* Visual Analog Scale, *MHQ* Michigan Hand Outcomes Questionnaire

### Methodological quality

The risk of bias assessment revealed significant methodological shortcomings across the included studies.

#### Randomized controlled trials (RCTs)

**Fig. 2:**
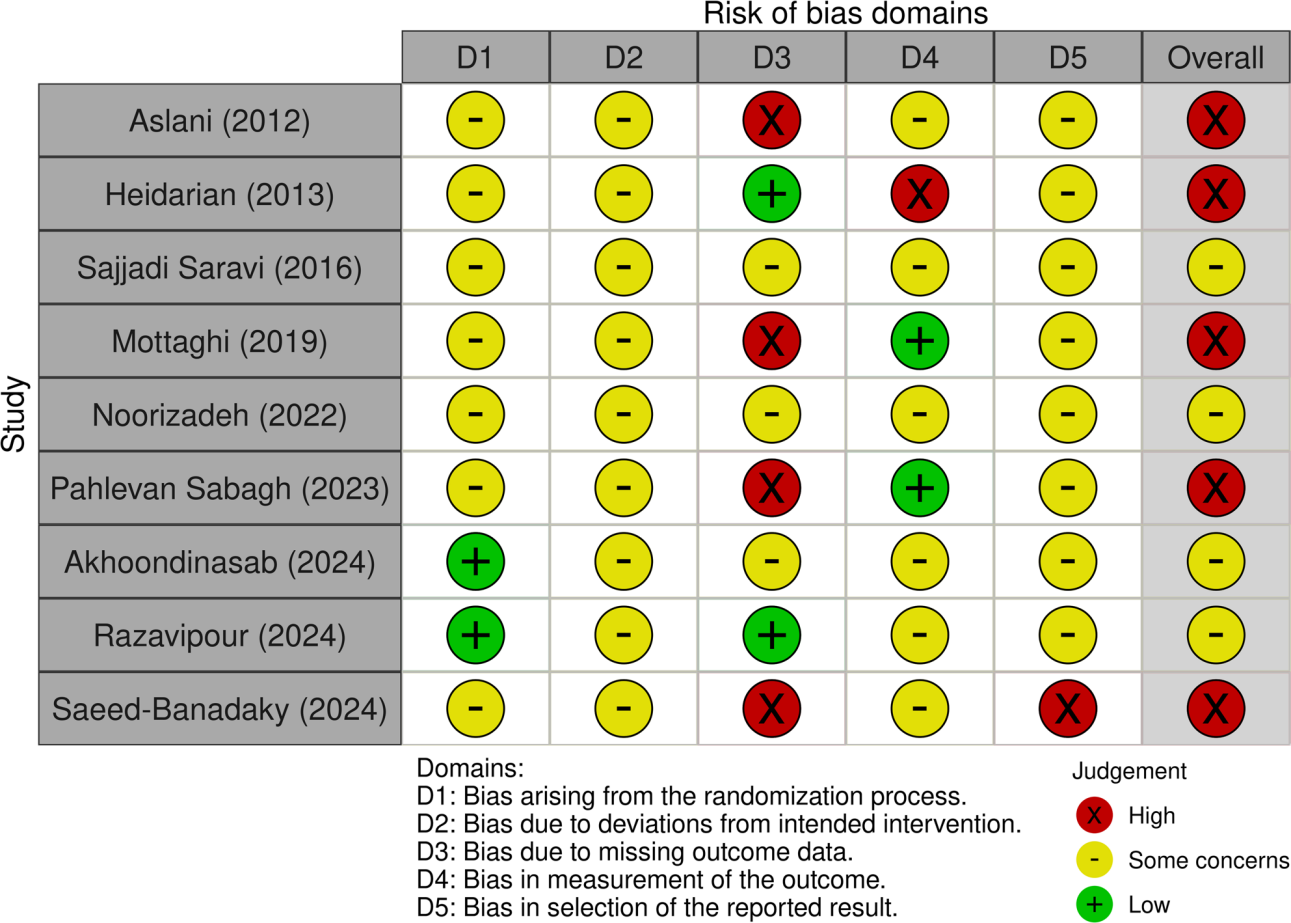
Risk of bias for included randomized controlled trials was assessed using the Cochrane Risk of Bias tool, RoB 2. Domains were: D1 (bias arising from the randomization process), D2 (bias due to deviations from intended interventions), D3 (bias due to missing outcome data), D4 (bias in measurement of the outcome), and D5 (bias in selection of the reported result). Judgement is color-coded as follows: green = low risk of bias, yellow = some concerns, and red = high risk of bias. Overall risk of bias was derived according to the RoB 2 algorithm based on domain-level judgements

The risk of bias for the 9 included RCTs was assessed using the Cochrane RoB 2 tool, as summarized in Fig. 2. None of the trials were judged to be at “low risk of bias” overall. Five studies (55.5%) were classified as “high risk, “primarily due to bias in missing outcome data (Domain 3) and issues with measurement of the outcome (Domain 4). The remaining four studies (44.4%) raised “some concerns,” often stemming from insufficient details regarding the randomization process or allocation concealment.

#### Non-randomized studies

Quality assessment of non-randomized studies is presented in Table 2. Among the 9 studies evaluated via the Newcastle-Ottawa Scale (NOS), only two (Ebrahimzadeh et al. and Khodadadi et al.) demonstrated “good” methodological quality. The remaining studies were classified as “fair” or “poor,” often due to short follow-up durations or failure to control for confounding factors.

**Table 2:** Methodological quality of included observational studies (Cohort and Case-Control) assessed using the Newcastle-Ottawa Scale (NOS)

Study (First author, year)	Selection	Comparability	Outcome / Exposure	Overall Quality
Ebrahimzadeh (2013)	☆★★★	☆★	☆★★	**Good**
Roshanzamir (2016)	★★★★	☆★	☆☆★	**Poor**
Abedi (2018)	☆★★★	☆☆	☆☆★	**Poor**
Afshar (2020)	☆★★★	☆★	☆☆★	**Poor**
Alimohammadi (2020)	☆☆★★	☆★	☆★★	**Fair**
Khoshnevis (2020)	☆★★★	☆☆	★★★	**Poor**
Razavipour (2021)	☆★★★	☆☆	☆☆★	**Poor**
Hajibarati (2022)	☆★★★	☆☆	★★★	**Poor**
Khodadadi (2022)	☆★★★	☆★	☆★★	**Good**

★ = Point awarded; ☆ = Point not awarded. Selection: Maximum 4 stars; Comparability: Maximum 2 stars; Outcome (for Cohort) / Exposure (for Case-Control): Maximum 3 stars. Quality Thresholds: Scores of 7–9 were considered “Good”, 5–6 “Fair”, and < 5 “Poor” (adapted thresholds, or based on specific domain failures)

Critical appraisal of cross-sectional and case series studies is provided in Supplementary Table S2. These studies, assessed using JBI checklists, generally demonstrated “moderate” quality, with consistent reporting of participants and outcomes but frequent lack of clarity regarding statistical analysis methods.

### Outcome measures

#### Patient-reported outcome measures (PROMs)

The BCTQ was the most frequently used standardized instrument, utilized in 45.5% of the studies (*n* = 10). Other validated tools, including the DASH, QuickDASH, and Michigan Hand Outcomes Questionnaire (MHQ), were employed in only 18% of studies.

#### Pain and physical examination

Pain intensity was assessed using the VAS in 13 studies (59.1%). Objective functional metrics were less consistently reported; grip strength was evaluated in 40.9% of studies, while pinch strength was measured in only 13.6%.

#### Electrodiagnostic testing

Although 81.8% of studies utilized electrodiagnostic testing (EMG-NCV) for preoperative diagnosis, only 4 studies (18%) assessed postoperative electrophysiological changes as an outcome measure.

Notably, none of the included studies reported interpretive thresholds such as the Minimal Clinically Important Difference (MCID) or Patient Acceptable Symptom State (PASS), relying solely on statistical significance (P-values) to demonstrate treatment efficacy.

### Surgical techniques and functional outcomes

Surgical techniques varied across studies, with the mini-incision technique being the most reported approach (*n* = 9). Critical functional milestones such as return to work and daily activities were reported in only five studies (22.7%). Additionally, postoperative scar length was documented in just 18% of the reviewed literature.

### Trends in outcome reporting

A temporal analysis of the included studies reveals an improving trend in the utilization of standardized outcome measures. Studies published from 2020 onwards demonstrated a higher adherence to standardized reporting compared to older publications, with a notable increase in the usage of the BCTQ and VAS. However, the comprehensive reporting of functional metrics, such as return to work time and complications, remained inconsistent across both time periods.

## Discussion

### Study design and evidence quality

The present systematic review provides a comprehensive analysis of outcome reporting in Iranian carpal tunnel surgery research. A primary finding is the predominance of observational designs, with RCTs constituting only 40.9% of the included studies. This proportion highlights a critical gap, as international standards are shifting towards high-quality RCTs to define surgical indications. For instance, the 2025 AAOS Clinical Practice Guideline relies heavily on high-quality RCTs to recommend surgical intervention over conservative management [29] .The reliance on lower-level evidence (cohort and descriptive studies) in the Iranian literature may compromise the validity of findings and their integration into global meta-analyses.

Our quality assessment indicates that the majority of Iranian RCTs suffer from high risk of bias, particularly in outcome measurement and reporting selection. This introduces a significant risk of overestimating treatment effects. Furthermore, the predominance of ‘Poor’ quality cohort studies suggests that the reported outcomes may not be generalizable or free from confounding factors. Future studies must adhere strictly to CONSORT guidelines for RCTs and STROBE guidelines for observational studies to improve internal validity.

### Standardization of patient-reported outcomes

In clinical research, standardized questionnaires are commonly employed to evaluate treatment outcomes. For upper limb surgeries, instruments such as the Disabilities of the Arm, Shoulder and Hand (DASH), its shorter version QuickDASH, and the Michigan Hand Outcomes Questionnaire are frequently used globally [30]. Our analysis showed that while 45.5% of Iranian studies utilized the BCTQ, this rate is significantly lower than international benchmarks. For instance, systematic reviews by Sousa et al. [31] and Mertz et al. [6] reported BCTQ usage rates of 75% and 60%, respectively. However, our temporal trend analysis suggests a positive shift: studies published after 2020 demonstrated a higher adherence to standardized reporting compared to older publications. Despite this, other critical domains such as sleep quality remain largely unassessed. A 2024 meta-analysis highlighted that sleep disturbance is a primary driver for seeking surgery [32], yet this variable was virtually absent in the reviewed Iranian studies.

### The clinical relevance gap

A critical shortcoming identified in this review is the absence of interpretive metrics such as the MCID and PASS. While Iranian studies consistently reported statistical significance (P-values), recent guidelines emphasize that statistical difference does not always equate to clinical benefit [29]. The lack of MCID reporting limits the ability to distinguish between trivial statistical changes and meaningful clinical improvements, hindering the application of value-based medicine. Future Iranian studies should strictly adopt the reporting of MCID to transition from statistical significance (P-value driven) to clinical relevance (Patient-centric).

### Objective and neurophysiological outcomes

Pain assessment using the VAS was reported in 60% of Iranian studies, which is comparable to the 51% reported in the systematic review by Mertz et al. [6]. However, objective functional metrics showed significant gaps. Thenar atrophy is a definitive sign of severe motor denervation and a primary indication for surgery [29]. Despite its clinical importance, muscle weakness, measured through pinch strength, grip strength, thenar atrophy, or thumb opposition, was assessed in only 9 studies (40.9%). This inconsistency aligns with findings by Geere et al. [33], who highlighted the lack of consensus on motor outcome assessment in the global literature. Furthermore, while 81.8% of studies utilized EMG-NCV for preoperative diagnosis, consistent with other systematic reviews [31], only 18% assessed postoperative neurophysiological changes. This suggests that in the Iranian orthopedic community, EDX is primarily viewed as a diagnostic tool rather than an outcome measure, contradicting clinical guidelines that suggest it for monitoring recovery in complex cases. Other aspects, such as sensory evaluation, were addressed in only 3 studies. This suggests a disconnect where Iranian surgeons prioritize subjective symptom relief over objective neurophysiological and motor recovery in the postoperative setting.

### Functional recovery and socioeconomic impact

Return to work and daily activity resumption are critical indicators of recovery. These variables were reported in only 22.7% of Iranian studies, which is notably lower than the 40% reported in the systematic review by Liao et al. [34].

A recent meta-analysis from 2024 put the global prevalence of carpal tunnel syndrome (CTS) at 14.4% [2].Beyond its clinical manifestations, CTS has been consistently characterized as a condition with a substantial socioeconomic burden, encompassing both healthcare-system and work associated consequences [35]. Specifically, evaluations of carpal tunnel release reveal that the societal impact of CTS encompass not only treatment costs but also significant indirect expenses related to lost work hours and diminished productivity [36].Consequently, current cost-effectiveness analyses of CTS therapies generally include direct medical expenses (such as surgery and rehabilitation) along with indirect costs, particularly absenteeism, highlighting their significant contribution to the total economic burden [37].

The lack of cost-effectiveness and return-to-work data in Iranian literature, given this considerable economic impact, constitutes a notable oversight. Within the context of Iran’s healthcare system and economic environment, understanding the cost-benefit ratios of various surgical techniques is essential for improving resource allocation. Future research in Iran should emphasize health economics by incorporating cost-effectiveness evaluations and diligently monitoring the timing for patients’ reintegration into the workforce and everyday routines. Moreover, only 4 studies (18%) of included studies evaluated surgical scar length. Hammert et al. [38], observed that scar length is substantially correlated with pillar discomfort; hence, its underreporting undermines the clinical significance of safety results. Surgical factors, including suture technique and local anesthetic use, were reported inconsistently among trials, complicating the correlation between surgical subtleties and clinical results. Finally, surgical variables such as suture technique and the use of local anesthesia were inconsistently reported across studies, making it difficult to correlate surgical nuances with clinical outcomes.

### Recommendations for future research

To enhance the comparability and therapeutic utility of studies on carpal tunnel release, future research should reduce outcome heterogeneity through the use of a concise, standardized outcome set with clear reporting of instruments, time points, and clinically interpretable estimates. We recommend: (i) the routine utilization of validated PROMs, specifically, the BCTQ as the primary measure for CTS and the DASH or QuickDASH for upper-limb disability, reported with clear scoring and validated language versions; (ii) the establishment of predefined follow-up intervals (e.g., early, mid-term, and ≥ 6 months) accompanied by explicit attrition reporting; (iii) the presentation of effect sizes with confidence intervals and, where applicable, clinically significant change thresholds; and (iv) the incorporation of outcomes frequently overlooked yet highly pertinent to patients and healthcare systems, particularly return-to-work rates, pillar pain, scar-related symptoms, and standardized complications. Considering the possible productivity losses and limited resources, Iranian research should integrate fundamental resource use and cost metrics, facilitating practical cost-effectiveness evaluations in conjunction with therapeutic outcomes.

### Limitations

This review has certain limitations. First, restricting eligibility to English-language publications to ensure international comparability may have introduced language bias. Although the review focused on Iranian populations, Persian-language databases were not searched and Persian-language studies were excluded, which may have led to the omission of relevant national evidence. Second, heterogeneity in surgical techniques and follow-up durations precluded a quantitative meta-analysis of clinical outcomes. Additionally, the review protocol was not prospectively registered in PROSPERO, which may reduce transparency and increase the risk of reporting bias. Consequently, the findings may not fully represent the entire body of Iranian research, but rather the portion accessible through international, English-indexed sources. Future studies are encouraged to incorporate validated questionnaires such as DASH and the Boston CTS Questionnaire. Furthermore, precise documentation of variables including anesthesia type, suture technique, treatment costs, and return-to-work time may enhance reporting quality, facilitate analyses, and improve applicability of results.

## Conclusions

Our findings suggest that the Iranian literature on carpal tunnel release is constrained not by a lack of data, but by uneven outcome selection and inadequate reporting, which collectively hinder synthesis, benchmarking, and practical application. In several studies, validated PROMs are applied inconsistently, follow-up intervals are poorly specified, and findings are frequently presented without clinically interpretable summaries, diminishing the evidence’s utility for clinicians and patients. Furthermore, outcomes that immediately indicate recovery and socioeconomic effects are often neglected, such as return-to-work status, job capability, symptoms linked to scar and pillar pain, and systematic complication reporting. These constraints, along with inconsistent study quality, emphasize the necessity for national conformity with worldwide result standardization initiatives. We recommend that future Iranian studies prioritize a succinct core outcome set (BCTQ and DASH), prespecify follow-up intervals, report effect sizes with confidence intervals, and consistently capture functional and safety outcomes, supplemented by fundamental resource-use metrics to facilitate locally pertinent economic evaluation. 

## Supplementary Information

Supplementary Material 1.

Supplementary Material 2.

## Data Availability

All data analyzed during this study are included in this published article. Additional details are available from the corresponding author upon reasonable request.

## Abbreviations

AAOS: American Academy of Orthopedic Surgeons
BCTQ: Boston Carpal Tunnel Questionnaire
CONSORT: Consolidated Standards of Reporting Trials
CTS: Carpal Tunnel Syndrome
DASH: Disabilities of the Arm, Shoulder and Hand
DML: Distal Motor Latency
DSL: Distal Sensory Latency
EDX: Electrodiagnostic
EMG-NCV: Electromyography and Nerve Conduction Velocity
JBI: Joanna Briggs Institute
MCID: Minimal Clinically Important Difference
MeSH: Medical Subject Headings
MHQ: Michigan Hand Outcomes Questionnaire
NCS: Nerve Conduction Study
NOS: Newcastle-Ottawa Scale
PASS: Patient Acceptable Symptom State
PRISMA: Preferred Reporting Items for Systematic Reviews and Meta-Analyses
PROMs: Patient-Reported Outcome Measures
RCT: Randomized Controlled Trial
RoB 2: Cochrane Risk of Bias tool (version 2)
SCV: Sensory Conduction Velocity
SD: Standard Deviation
STROBE: Strengthening the Reporting of Observational Studies in Epidemiology
VAS: Visual Analog Scale
