# Supplementary material for: “Outcome measures in Iranian studies of carpal tunnel syndrome surgery: a systematic review”
Source: BMC Surg. 2026 Apr 27;26:415. doi: 10.1186/s12893-026-03712-7 (PMC13270547; doi:10.1186/s12893-026-03712-7)
Supplement: Supplementary file 1 — Supplementary Material 1. [file 12893_2026_3712_MOESM1_ESM.docx]

| **Study (First author, year)** | **Q1** | **Q2** | **Q3** | **Q4** | **Q5** | **Q6** | **Q7** | **Q8** | **Q9** | **Q10** | **Overall Quality** |
| --- | --- | --- | --- | --- | --- | --- | --- | --- | --- | --- | --- |
| **Tahririan (2012)** | Yes | Yes | Yes | Unclear | Unclear | Yes | Yes | Yes | No | Yes | **Moderate** |
| **Nazerani (2014)** | Yes | Yes | Yes | Unclear | Yes | Yes | Yes | Yes | Yes | Yes | **Good** |
| **Mardanpour (2019)** | Yes | Yes | Yes | Unclear | Unclear | Yes | Unclear | Yes | Yes | Unclear | **Moderate** |
| **Daliri (2022)** | Yes | Yes | Unclear | Yes | No | No | Yes | Unclear | N/A | N/A | **Moderate** |

***Supplementary Table S2***. Critical appraisal of included case series and cross-sectional studies using the JBI Checklist.
Legend: Q1: Criteria for inclusion clearly defined; Q2: Study subject and setting described in detail; Q3: Exposure measured in valid or reliable way; Q4: Objective, standard criteria used for measurement of condition; Q5: Confounding factors identified; Q6: Strategies to deal with confounding factors stated; Q7: Outcomes measured in valid or reliable way; Q8: Appropriate statistical analysis used; Q9: Comparison group appropriate (if applicable); Q10: Groups comparable (if applicable). N/A: Not Applicable.
